# Geographical distribution of close kin in southern right whales on feeding grounds

**DOI:** 10.1371/journal.pone.0301588

**Published:** 2024-04-25

**Authors:** Megumi Takahashi, Brage Førland, Luis A. Pastene, Hans J. Skaug

**Affiliations:** 1 Institute of Cetacean Research, Chuo-ku, Tokyo, Japan; 2 Department of Mathematics, University of Bergen, Bergen, Norway; 3 Project R20F0009, Centro de Estudios del Cuaternario de Fuego, Patagonia y Antártica (CEQUA), Punta Arenas, Chile; 4 Institute of Marine Research, Nordnes, Bergen, Norway; MARE – Marine and Environmental Sciences Centre, PORTUGAL

## Abstract

This study investigated the close kinship structure of southern right whales on feeding grounds during austral summer seasons. The study was based on biopsy samples of 171 individual whales, which were genotyped with 14 microsatellite DNA loci. Kinship was investigated by using the LOD (Log Odds) score, a relatedness index for a pair of genotypes. Based on a cut-off point of LOD_PO_ > 6, which was chosen to balance false positives and negatives, a total of 28 dyads were inferred. Among these, 25 were classified as parent-offspring pairs. Additional genetic (mitochondrial DNA haplotypes) and biological (estimated body length, sex) data were used to provide additional information on the inferred close kin pairs. The elapsed time between sampling varied from 0 (close kin detected in the same austral summer season) to 17 years. All the kin pairs occurred within the Antarctic Indo sector (85°-135°E) and no pair occurred between whales within and outside of this sector. Six pairs were between individuals in high (Antarctic) and lower latitudes. Results of the present analysis on kinship are consistent with the views that whales in the Indo sector of the Antarctic are related with the breeding ground in Southwest Australia, and that whales from this population can occupy different feeding grounds. The present study has the potential to contribute to the conservation of the southern right whales through the monitoring of important population parameters such as population sizes and growth rate, in addition to assist the interpretation of stock structure derived from standard population genetic analyses.

## Introduction

**Fig 1:**
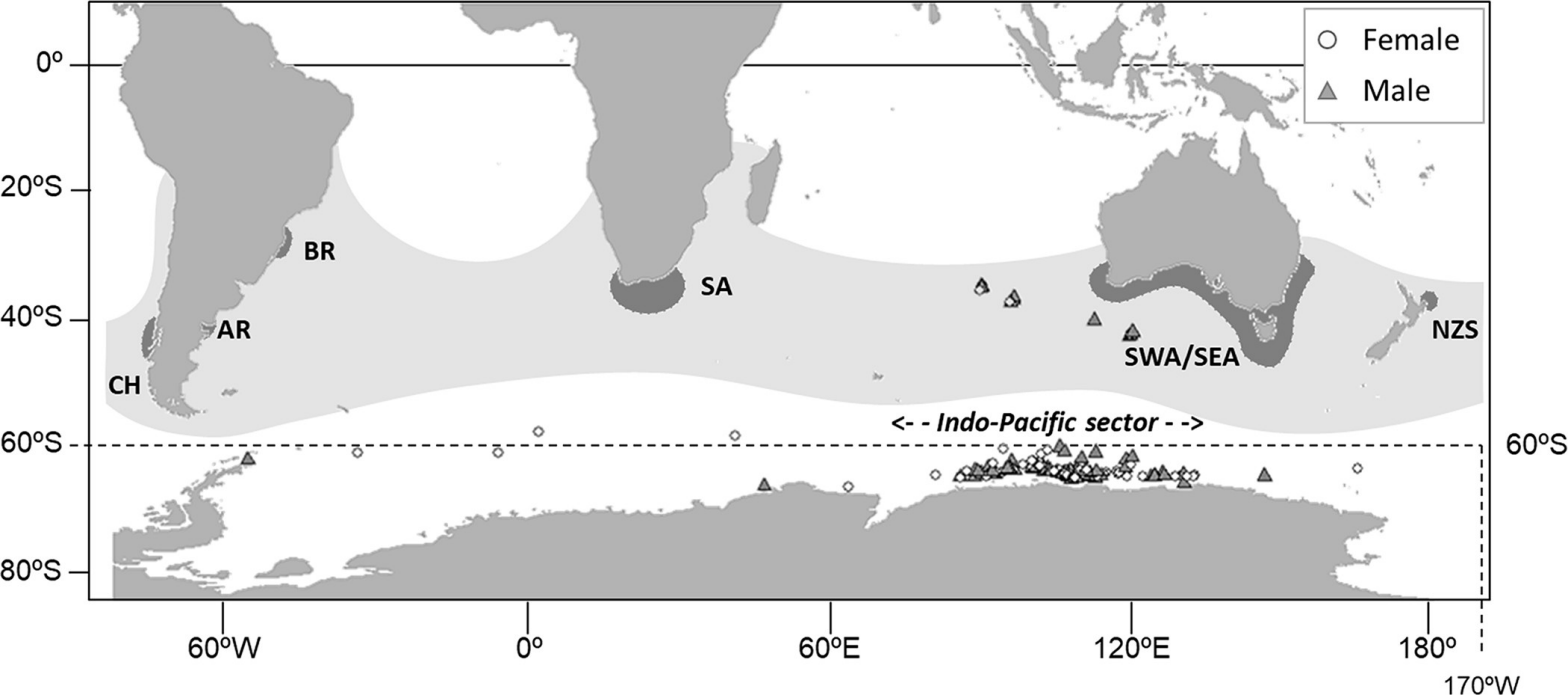
Historical distribution and primary calving grounds of southern right whales. The geographical positions of the sampled whales are shown by sex. Surveys were conducted in the austral summer seasons (December to March) from 1993/94 to 2018/19. The map was obtained in the R statistical environment [6] using *rnaturalearth* package [7], and the world vector map data was obtained from the Natural Earth (public domain): http://www.naturalearthdata.com.

Southern right whales (*Eubalaena australis*) are widely distributed across the three ocean basins in the Southern Hemisphere: South Atlantic, Indian Ocean, and South Pacific, mainly between latitude 16°S and 65°S. Southern right whales approach the continental coasts and some islands for breeding, calving, and resting during the austral winter and early spring. The primary breeding grounds of this species are located in the waters off South Africa, South West Australia, mainland New Zealand, New Zealand Sub-Antarctic and Argentina (Fig 1) [1–5]. Also, southern right whales occur in winter in coastal areas of South Eastern Australia, Chile and Brazil.

Southern right whales were heavily exploited by commercial whaling (Yankee-type whaling) during the 18^th^ and 19^th^ centuries. As a consequence, their abundance decreased to levels near extinction. Fortunately, some studies have reported an increase in the number of animals in some populations in recent decades [8–11]. Pre-exploitation abundance was estimated at 70,000–100,000 animals in the Southern Hemisphere [5,12]. The IWC Scientific Committee (IWC SC) reported that several populations e.g., the best known off Argentina, Australia and South Africa are increasing at annual rates of the order of 7–8% with an estimated total abundance of about 7,000 [4]. Also, the IWC SC showed evidence that the New Zealand sub-Antarctic population has increased. By 2009 the total population on the calving grounds was around 14,000 animals [5]. The IWC SC also noted that populations are recovering at different rates, and that low abundances persist in some population such as Brazil, southeastern Australia and Chile-Peru, and that demographic changes have been observed in South Africa since 2015 [13]. Continued monitoring of this recovery is required.

The research effort to investigate stock structure, distribution and trends in abundance has focused mainly on the breeding grounds, and limited information exists on feeding ground distribution, site fidelity and movements, and on the connection between breeding grounds in low latitude waters and higher latitude feeding grounds. Some of those studies focused on South Georgia [14–16]. Telemetry has been used recently to investigate movement patterns of South African [17] and western South Atlantic [18,19] right whales. The telemetry and photo-identification (e.g., [20]) studies in those ocean basins showed that southern right whales are found throughout large areas of the South Atlantic Ocean and visit several potential feeding areas each season. Furthermore, southern right whales from the Argentinean and South African wintering grounds may mix and potentially mate on shared feeding grounds [21].

The present study is based mainly on the Indian sector of the Antarctic. Previous studies based on distribution of historical whaling data, sighting surveys and photo-id have shown that animals from this Antarctic sector are associated with breeding grounds in the Australasian region [4,5,22]. Bannister et al. [23] reported photo-id matches between whales identified off either Western Australia or South Australia in winter/spring with whales in waters around 40°-44°S; 116°-125°E where a sighting of 35 animals were observed in December-January 1995–96. A southern right whale photographed in the Antarctic Indo sector in February 1996 had been identified over a period of 18 years on the coast of Western Australia [24].

Telemetry data suggested three probable foraging grounds for the animals tagged in the Australasian wintering grounds, to the southwest of Western Australia, the Subtropical Front and Antarctic waters [25], which is consistent with the sighting and photo-id data. Furthermore, these authors suggested that the observed variable population growth rates between wintering grounds in Australasian waters might reflect fidelity to different quality feeding grounds. On the other hand, Pastene et al. [26] conducted a study of individual identification based on microsatellite DNA genotyping, and showed that at least some females and males visit the same feeding ground in different years in the Indian sector of the Antarctic south of 60°S in the austral summer.

In this study, the geographical distribution of close kin in southern right whales is investigated based on an extended sample set of that used by Pastene et al. [26], which involves 183 biopsy samples obtained mainly in the Antarctic longitudinal sector 85°-135°E in the period 1993–2019. The genetic determination of close kin pairs has been proposed as a general framework for estimating key population parameters, such as population size and growth rate [27]. Also, kinship information can assist the interpretation of movement and social and population structure [28]. Therefore, the present study has the potential to contribute to the conservation of the southern right whales through the monitoring of important population parameters.

## Materials and methods

### Samples

A total of 183 skin/blubber biopsy samples were obtained opportunistically from free-ranging southern right whales along the sighting surveys of the Japanese Whale Research Program under Special Permit in the Antarctic (JARPA/JARPAII), the International Whaling Commission-International Decade for Cetacean Research/Southern Ocean Whale and Ecosystem Research (IWC-IDCR/SOWER) program, and the New Scientific Whale Research Program in the Antarctic Ocean (NEWREP-A) in the Antarctic Ocean from 60°W to 170°W across the 180° meridian, south of 35°S during the austral summer seasons (December to March) 1993/94 to 2018/19. Most of the samples were taken in the Antarctic Indian sector (85°–135°E), south of 60°S from 1993/94 to 2015/16. To get the genetic samples, various biopsy systems were used, including crossbows, air guns (e.g., [29,30]) and modified shotguns (Larsen, 1998 [Unpublished]), all using modified collection darts that took a small sample of skin and blubber. These biopsy samples obtained in the high seas of international waters by Japanese dedicated sighting surveys were authorized by annual SUIKAN permits issued by the Fisheries Agency, Government of Japan. Most of the 183 samples were used in a previous study on genetic individual matching [26]. The geographical distribution of the southern right whales sampled is shown in Fig 1. For each sample, sampling date and geographical location was available. In some cases, visually estimated measurements of body lengths of the animals sampled were recorded, in conjunction with other ancillary information such as the presence of calf. Visual estimates of body length were made from the vessels by experienced scientists and crew members. This additional non-genetic information assisted the interpretation of the results of close kinship inferences. See S1 Table for the whole data set used in the present study.

### DNA profile preparation

The laboratory procedures used to generate the microsatellite DNA and mtDNA data were very similar to those used in a study on North Pacific right whales by the same laboratory [31]. The only difference was the number of microsatellite loci used. The main steps of the procedures are repeated here. Genomic DNA was extracted from approximately 0.05 g of the outer epidermal layer of the skin tissue using standard phenol-chloroform protocols [32] or using Gentra Puregene kits (QIAGEN). Extracted DNA was stored in TE buffer (10 mM Tris-HCl, 1 mM EDTA, pH 8.0).

The DNA samples were genotyped using 14 microsatellite loci: EV1*Pm*, EV14*Pm*, EV21*Pm*, EV37*Mn*, EV94*Mn* [33], GT023, GT211, GT310 [34], GATA028 [35], DlrFCB17 [36], TR3G2, TR2G5, TR2F2, and TR3F3 [37]. The SRY locus located on the Y chromosome was also used for sex determination following the method of Abe et al. [38] with a slight modification informed in Pastene et al. [31].

Locus amplification followed those of the original authors. PCR amplifications were performed in 15 μl reaction mixtures containing 10–100 ng of DNA, 0.25 μM of each primer, 0.35 units of Ex Taq DNA polymerase (Takara), and 0.2 mM of dNTPs, and 1x Ex Taq buffer (Mg^2+^ plus) (Takara). All PCR products were electrophoresed on an Applied Biosystems 3500 Genetic Analyzer. Allele sizes were determined using a GS-600 LIZ size standard and GeneMapper v. 5.0 (ABI) software. The computer program MICRO-CHECKER [39] was used to check for null alleles and reading/typing errors for the microsatellite data. All samples were genotypes at all loci. Data were missed for only one sample at two loci.

The first 470 base pairs (bp) at the 5’ end of the mtDNA control region were amplified by polymerase chain reaction (PCR) using primers MT4 [40] and Dlp5R (5’-CCA-TCG-AGA-TGT-CTT-ATT-TAA-GGG-GAA-C-3’). Specifications for the PCR and cycle sequencing reactions are provided in Pastene et al. [31]. Each sample was sequenced in both forward and reverse directions (i.e., using both primers). The program Sequence Navigator was used to edit the sequences and put the forward and reverse together into a consensus sequence. Cases of singletons (haplotypes found in a single individual) were confirmed by repeating the sequencing (in both directions) for the particular samples.

Microsatellite DNA genotypes were the primary data used for estimating close kinship. The microsatellite DNA genotypes will be available to interested scientists following requests to the Institute of Cetacean Research.

Mitochondrial DNA haplotypes and sex information were only used to assist the interpretation of close kinship inferences. The haplotypes used for interpretation purposes matched with the following sequences in GenBank: Haplotype 1 (access number: BakHapA (JN097593)), Haplotype 2 (BakHapBplus (JN097595)), Haplotype 3 (BakHapC (JN097596)), Haplotype 4 (BakHapD (JN097597)), Haplotype 5 (BakHapE (JN097598)), and Haplotype 14 (CarHapJ (JN097600)).

### Close kinship inference

The LOD (Log Odds) score, a relatedness index for a pair of genotypes [28], was generated for all pairs of individuals included in the study. The LOD is defined as the log-likelihood ratio of the probability of an observed genotype pair given that they share a certain relationship, e.g., parent-offspring, relative to the probability of the genotype pair given that they are unrelated. For the parent-offspring relationship (PO), this amounts to

$$LOD_{PO}(i,j)=ln(\frac{P(G_{i},G_{j}|Parent-offspring)}{P(G_{i},G_{j}|Unrelated)}).$$

Here, *G*_*i*_ and *G*_*j*_ are the observed genotypes for two specimens, *i* and *j*. LOD scores for other kinship categories are defined similarly. The observed genotypes may contain genotyping errors. In this study, a simple error model with a constant and independent per-allele error rate, was used. For a parent-offspring pair and self/monozygotic relationships, an error model ensures that the LOD score is well defined for all genotype pairs; otherwise, the LOD score would be undefined for all pairs containing loci with no compatible alleles. The second-degree relationships of half-siblings (HS), grandparents, and full aunts and uncles all have the same LOD score and cannot be separated on basis of genotypes. We refer to this as the LOD_HS_ (half-sibling LOD score). It should be kept in mind that LOD_HS_ does not necessarily refer to half-siblings alone. We define the maximum likelihood kinship for a genotype pair as the kinship category that gives the highest probability for the observed genotype, i.e., the relationship with the highest $P(G_{i},G_{j}|\mathrm{Kinship})$. This is equivalent to selecting the kinship category with the highest LOD score, since its denominator is the same for all kinship categories.

LOD scores were calculated using the R package CKMRsim [41], which has a flexible genotyping error model built in. Further, CKMRsim can simulate the LOD score distribution in populations consisting entirely of a specific kinship category. Using CKMRsim, we simulated three datasets, each consisting of 10^6^ genotype pairs (*G_i_, G_j_*), for the relationships PO (parent-offspring), HS (“half-sibling”), and U (unrelated), using the allele frequencies calculated from the sample, and an assumed genotyping error rate (epsilon) of 0.0077/allele. This genotyping error rate per allele was based on the value for North Pacific right whales used by Pastene et al. [31]. For each genotype pair in the simulated datasets, we calculated LOD_HS_ and LOD_PO_. This provided the frequency distribution for both LOD scores, each for the three simulated datasets. The separation of the three distributions for LOD_PO_, say, determines the ability of LOD_PO_ to discriminate between different kinship categories. If the LOD distributions are close or overlapping, setting a LOD cut-off value will determine the expected number of false positives and false negatives. Depending on the purpose of the classification, a high cut-off value might be set to get a smaller sample of close kin pairs with high certainty, or a lower cut-off value could be set to get a larger sample of less certain pairs.

The kinship category (PO, FS, HS or U) with the highest LOD score was assigned as the maximum likelihood kinship, where FS denotes full siblings. The false positive rate for parent-offspring pairs at different LOD cut-off thresholds was estimated from the simulated unrelated pairs classified as parent-offspring pairs at each cut-off value. The false negative rate was similarly estimated using the simulated parent-offspring distributions from the number of pairs at each cut-off value which were misclassified. The LOD scores for the real data were calculated using CKMRsim with the same configuration as for the simulated data.

### Relationship between close kinship and geographical distance

To test the hypothesis that closely related individuals have a geographical association, two different statistical methods were applied to data consisting of inferred PO pairs. Firstly, a standard two-sample Wilcoxon (nonparametric) test was applied to a dataset of the difference (Δ) in longitude, separating two individuals. Throughout, we use Δ longitude, rather than geographical distance because the main interest in this study was the investigation of longitudinal expansion of the population. The data set consisted of all 14,535 pairwise comparisons of all individuals (171, see below) examined (excluding pairs observed as ’mother and calf pair’ by direct observation in the field, see below). The two groups in the Wilcoxon test were those assigned as PO pairs (*n* = 60) and unrelated (*n* = 12,223) by maximum likelihood classification. Pairs in the HS category were not used in the Wilcoxon test. We did not use cut-off level for LOD_PO_ in this part of our study, i.e. the *n* = 60 PO pairs were those classified by maximum likelihood in the full set of 14,535 pairwise comparisons. This was done to balance the two factors that affect statistical power: sample size and signal strength, with the latter being somewhat diluted by the introduction of false positives into the PO group.

The reason for using a nonparametric Wilcoxon test instead of a t-test is that Δ longitude is not normally distributed within PO pairs. The 12,283 pairwise comparisons will not be fully statistically independent because each individual contributes to 171–1 = 170 pairwise comparisons. In principle, such dependence could affect the significance level of the test, but simulation experiments in a similar setting have shown that this is not a major concern [28]. Similarly, we applied the Wilcoxon test restricted to the Antarctic Indo sector where most sighting and biopsy samples of this species are concentrated. The two groups in this test were those assigned as parent-offspring (*n* = 58) and unrelated (*n* = 10,801), respectively.

Secondly, linear quantile regression [42] was applied, with LOD_HS_ as the response variable. This technique consists of regressing the τ-quantile in the LOD score distribution on one or more explanatory variables, with the rationale being that the close-kin pairs are found in the very right-hand tail (τ close to 1) of the LOD score distribution. We used two explanatory variables: Δ longitude and SameDayPos, with the latter being a dichotomous categorical measure of sampling proximity, i.e. whether or not the two individuals in a pair were sampled on the same day and by the same survey vessel. Including SameDayPos in the quantile regression amounts to splitting the dataset two, and comparing the τ quantiles of the resulting parts, for a range of τ values. Including a continuous covariate (Δ longitude) assumes a linear relationship between the covariate and the τ quantile in the LOD distribution. A negative slope of the regression line for Δ longitude would have the interpretation that close kin aggregate geographically. The quantile regression was done using the R-package ‘quantreg’ [43], with quantiles (τ) ranging from 0.900 to 0.999, and all statistical analyses in this study were done in R 4.2.3 [6].

## Results

### Genetic analysis

The number of alleles per microsatellite locus ranged from 2 to 15. There was no significant deviation from the Hardy-Weinberg genotypic proportion. The genotypic analyses revealed four cases of duplicates (two samples obtained from the same individual at the same location and time). It also revealed eight cases of individuals re-sampled in different locations and time. Only one of the duplicate and re-sampled individuals were used in the present analysis. Then the total number of individual samples used in the present analysis was 183–12 = 171.

The genetic-based sex determination showed 86 females and 85 males in the total sample. The mtDNA analyses revealed a total of 8 haplotypes in the total sample. As noted earlier, sex determination and mtDNA haplotypes, as well other non-genetic information obtained at the time of the biopsy sampling, were used only to assist the interpretation of close kinship inferences.

### LOD score analyses

**Fig 2:**
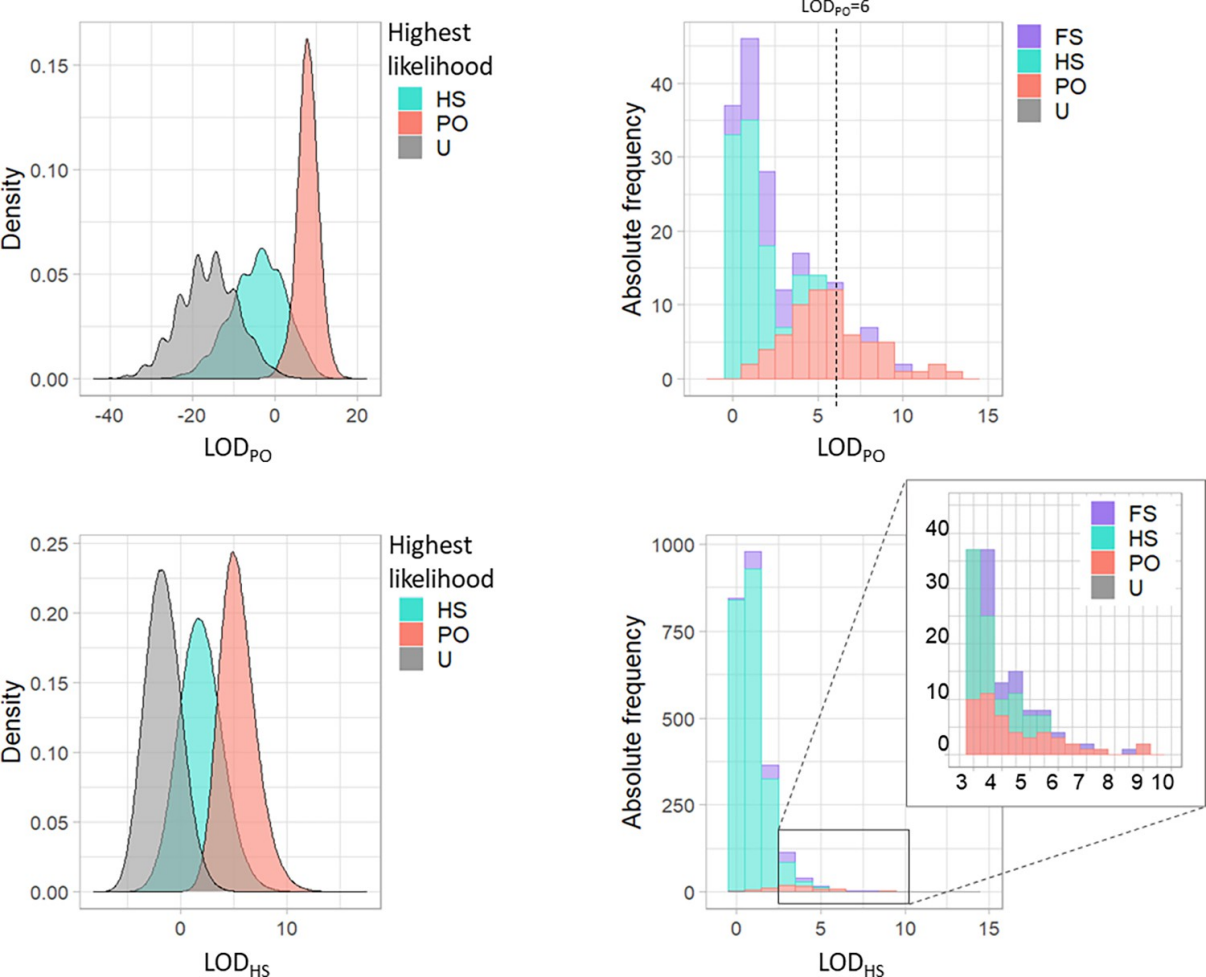
Simulated (left) and observed (right) distribution of the LOD_PO_ (top) and LOD_HS_ (bottom) scores. For the three simulated datasets (HS, PO, U) normalized densities (unit area) are shown, while for the real data the absolute frequency of each relationship category, as assigned by the maximum likelihood, is displayed.

Fig 2 shows the LOD score distributions (LOD_PO_ and LOD_HS_) for simulated and observed data sets. There was an overlap between the three distributions simulated from different kinship categories (Fig 2, left). This overlap prevents a perfect classification into kinship categories and motivates our use of maximum likelihood classification of kinship category. It should be noted that for comparison purposes the simulated distributions are normalized for each category separately. In a real population there are many more unrelated than related pairs, which effectively makes the distribution for the unrelated category overlap more with the two other distributions than is apparent from the normalized distribution. This is seen for the real data (Fig 2, right). The LOD_PO_ score for the simulated HS and unrelated datasets has multimodal distributions. Each mode corresponds to a specific number of loci having allelic values, for the two individuals in question, being inconsistent with a PO relationship (without allowing for genotyping error, all these pairs would have a LOD score of minus infinity.)

The right-hand side of Fig 2 shows the LOD score distribution (left truncated) using either LOD_PO_ or LOD_HS_. The data set is the 171 southern right whale DNA profiles that yielded a total of 14,535 pairwise LOD values. The LOD distribution is subdivided and colored according to which kinship category has the highest probability (maximum likelihood classification). It should be emphasized that this is a probabilistic classification, which will not be error-free. For instance, twins are rare [44,45], and we do not have any evidence of monogamy in this species, so FS can also be expected to be rare. Hence, although some pairs are classified as full siblings using the maximum likelihood classification, the full sibling distribution (not shown in Fig 2) has a large overlap with both the half sibling and parent-offspring distribution, and the evidence that they are true full sibling pairs is weak. It is therefore likely that the true relationship of pairs classified as full sibling is parent-offspring or half sibling. Further, as noted earlier, grandparent-offspring relationships have the same LOD score as the HS category. Hence, what we have labelled HS in both Fig 2 could contain some GP pairs.

Fig 2 is also informative about the differences between LOD_PO_ and LOD_HS_. For instance, in the upper right panel the PO pairs are found in the very right hand of the distribution, but in the bottom left panel they are shifted somewhat to the left. This is a natural consequence of the way the two LOD scores are constructed, but it illustrates that it matters which of LOD_PO_ and LOD_HS_ is used to screen which pairs of individuals should enter the maximum likelihood stage of the analysis.

Table 1 shows the number of detected close kin dyads at various levels of the cut-off point of LOD score, as well as the expected number of false positives from unrelated pairs. Here, the challenge is to find a way on how to determine the value of the cut-off point that will maximize the number of correctly inferred pairs of related individuals while keeping the number of pairs incorrectly inferred as close relatives to a reasonable minimum. According to previous studies, using the LOD_HS_ is better suited than LOD_PO_ as a general test aimed at detecting all types of close 1st- and 2nd-order relationships [28]. Note that it is possible to get an unbiased estimate of the true number of, for instance, PO pairs by taking the observed number in Table 1, subtracting the expected number of false positives, and adding the expected number of false negatives. This can be done at various cut-off levels for the LOD score. The cut-off point of >6 was determined in consideration of the estimation of the false positive rate derived from genotyping error, and on the statistical power for detecting close related individuals.

**Table 1:** Number of related pairs by different (maximum probability) kinship category (PO/HS/FS), for different cutoff LOD values.

Kinship categories for LOD score	Cutoff point	# highest likelihood (# expected FP)								# expected FN on category "PO"
		FS			HS			PO		
HS	8	1	-		-	-		2	-	-
	7	1	-		-	-		4	(0.01)	-
	6	2	(0.09)		-	(0.16)		8	(0.19)	-
	5	5	(1)		6	(1)		15	(0.97)	-
	4	9	(4)		14	(8)		25	(4)	-
	3	28	(18)		41	(49)		43	(12)	-
PO	8	2	(0.07)		-	-		13	(0.6)	15
	7	3	(0.16)		-	-		18	(1)	11
	6	3	(0.29)		-	(0.01)		25	(4)	8
	5	4	(0.74)		1	(0.31)		39	(9)	6
	4	6	(1)		4	(1)		51	(16)	4
	3	9	(3)		7	(4)		58	(23)	3

In parenthesis the expected numbers of false positives are shown.

### Genetic-based identification of close kin dyads

Table 2 shows the 28 dyads with LOD_PO_ > 6 (information on pairs with LOD_PO_ < 6 is presented in Table 2). They consist of a total of 25 possible PO pairs and three possible FS pairs, as determined by maximum likelihood classification. Some additional genetic and biological information is also presented in Table 2, which can assist in the interpretation of close kinship categories. Four pairs recorded as mother and calf by observations in the field were confirmed as such by the genetic analysis. Eight male-female PO (or classified as FS) pairs shared the same mtDNA haplotype. In three of those cases, females were larger than males, which is consistent with a mother-son relationship. Similarly, six female-female pairs shared mtDNA haplotype and were thereby consistent with a mother-daughter relationship. In three of those cases, body length information allowed the identification of the mother in the dyad.

**Table 2:** Summary of information for the 28 dyads (D1,D2) identified with LOD_PO_ > 6.

#	Threshold on LOD_PO_	ID		LOD score (14 loci)			mtDNA haplotype*		Sex		Kinship category**	Body length (m)		Field observation***	Sampling Date		Sampling location					
		D2	D1	Half-sibling	Parents-offspring	max. likelihood	D2	D1	D2	D1		D2	D1		D2	D1	D2 Lat.	D2 Long.		D1 Lat.	D1 Long.	
1		14AJ4R031	18ANR01	8.83	12.55	PO	1	1	F	M	MS	14.1	15.4		2015-02-27	2018-12-01	-65.13	108.86	E	***-37*.*67***	95.94	E
2		01IVR024	14AJ4R018	8.94	12.46	PO	3	2	M	M		13.2	14.2		2002-02-15	2015-02-25	-64.61	92.25	E	-64.49	106.46	E
3		14AJ4R023	14AJ4R024	7.68	11.63	PO	5	5	M	F	MS	10.3	14.2	MC	2015-02-25	2015-02-25	-64.61	106.79	E	-64.61	106.79	E
4		01IVR022	18ANR02	7.03	10.65	PO	3	1	M	F		13.8	14.8		2002-02-15	2018-12-01	-64.61	92.25	E	***-37*.*67***	95.94	E
5		01IVR016	01IVR017	6.50	9.59	PO	1	1	F	F	MD	13.6	10.0	MC	2002-02-14	2002-02-14	-64.47	94.05	E	-64.47	94.05	E
6		97IVR008	98SWR137	8.34	9.56	FS (PO)	-	1	M	F		12.8	-		1998-01-21	1999-02-02	-65.08	112.93	E	-63.00	100.83	E
7		07SWR017	18ANR03	6.47	9.31	PO	4	4	F	F	MD	-	12.5		2008-02-10	2018-12-05	-64.75	106.19	E	***-35*.*01***	90.36	E
8		14AJ4R038	14AJ4R039	6.08	9.29	PO	2	2	F	F	MD	10.2	14.3	MC	2015-02-28	2015-02-28	-63.92	111.28	E	-63.92	111.28	E
9		05IVR36	98SWR041	6.14	8.94	PO	2	2	M	M		16.6	-		2006-02-21	1999-01-28	-62.17	110.01	E	-63.00	95.85	E
10		05IVR44	93IVR003	5.57	8.69	PO	3	-	F	M		13.0	13.7		2006-03-12	1994-03-05	-65.01	128.74	E	-64.23	113.07	E
11		01IVR027	09SWR016	5.58	8.68	PO	3	3	F	M	MS	13.5	-		2002-02-17	2010-01-30	-64.09	87.34	E	-64.80	107.19	E
12		09SWR024	18ANR05	5.82	8.47	PO	2	1	M	M		-	13.8		2010-02-03	2018-12-05	-63.51	100.18	E	***-35*.*14***	90.32	E
13		14AJ4R005	14AJ4R007	5.33	8.30	PO	1	1	F	F	MD	13.4	13.1		2015-02-22	2015-02-23	-62.48	100.21	E	-60.92	103.50	E
14		01IVR014	07IVR52	5.76	8.25	FS (PO)	2	2	F	M	MS	17.0	15.0		2002-01-11	2008-02-22	-61.41	102.20	E	-63.89	95.83	E
15	> 8	07SWR017	11AJ5R001	5.02	8.04	PO	4	4	F	F	MD	-	12.1		2008-02-10	2012-02-28	-64.75	106.19	E	-64.85	132.62	E
16		05IVR45	93IVR003	4.92	7.73	PO	1	-	M	M		13.3	13.7		2006-03-15	1994-03-05	-64.87	124.53	E	-64.23	113.07	E
17		07IVR60	14AJ4R034	5.06	7.60	PO	2	2	M	F	MS	13.0	12.7		2008-03-04	2015-02-27	-64.14	92.37	E	-64.18	110.92	E
18		09SWR023	16ANR01	5.03	7.54	FS (PO)	2	2	M	F	MS	-	14.8		2010-02-03	2017-01-14	-63.51	100.18	E	-63.87	164.92	E
19		05IVR38	99IVR011	4.68	7.37	PO	5	2	F	M		13.5	12.5		2006-03-04	2000-01-20	-63.68	102.40	E	-61.25	112.88	E
20		07IVR48	07VR105	4.25	7.29	PO	3	4	M	M		12.8	16.0		2008-02-22	2008-03-12	-63.67	95.81	E	-65.98	130.39	E
21	> 7	93IVR004	98SWR147	4.69	7.03	PO	3	3	M	F	MS	14.3	-		1994-03-05	1999-02-11	-64.23	113.07	E	-64.20	117.24	E
22		07SWR011	97IVR007	4.49	6.80	PO	5	1	M	M		-	11.9		2008-02-04	1998-01-15	-65.45	107.83	E	-62.92	100.47	E
23		09SWR012	14AJ4R037	3.93	6.74	PO	1	3	F	M		-	13.7		2010-01-30	2015-02-28	-64.57	107.77	E	-64.08	111.03	E
24		14AJ4R013	97IVR009	4.37	6.71	PO	14	-	F	M		12.2	13.4		2015-02-24	1998-02-10	-64.18	103.20	E	-61.09	106.74	E
25		05IVR34	05IVR41	4.07	6.46	PO	1	1	F	M	MS	16.3	14.5		2006-02-04	2006-03-06	-65.10	86.08	E	-64.02	107.76	E
26		14AJ4R006	14AJ4R007	4.21	6.44	PO	1	1	F	F	MD	10.5	13.1	MC	2015-02-23	2015-02-23	-60.92	103.50	E	-60.92	103.50	E
27		07IVR48	14AJ4R033	3.81	6.40	PO	3	1	M	F		12.8	14.2		2008-02-22	2015-02-27	-63.67	95.81	E	-64.60	110.41	E
28	> 6	05IVR34	07SWR001	4.17	6.28	PO	1	-	F	M		16.3	-		2006-02-04	2007-12-26	-65.10	86.08	E	***-40*.*49***	112.69	E

The second column shows the cut-off point for detecting the kinship by LOD score. Mitochondrial DNA haplotypes and other biological data are listed in the table, which assisted the interpretation of close kin relationships.

*: Numeral show mtDNA haplotypes. Nucleotide sequence of Haplotypes 1, 2, 3, 4, 5, and 14 are available in the DDBJ/EMBL/GenBank databases under the accession number(s) BakHapA (JN097593), BakHapBplus (JN097595), BakHapC (JN097596), BakHapD (JN097597), BakHapE (JN097598), and CarHapJ (JN097600), respectively.

**: This kinship category (MS: Mother and son, MD: Mother and daughter) is derived by mtDNA haplotype, and sex determination. The kinship category is not intended to reflect which individual is the presumed mother and which is the presumed offspring (i.e., in a kinship category of MD either individual D1 or D2 could be the inferred mother).

***: MC is abbreviation of Mother and Calf which is recognized on field observations.

**Fig 3:**
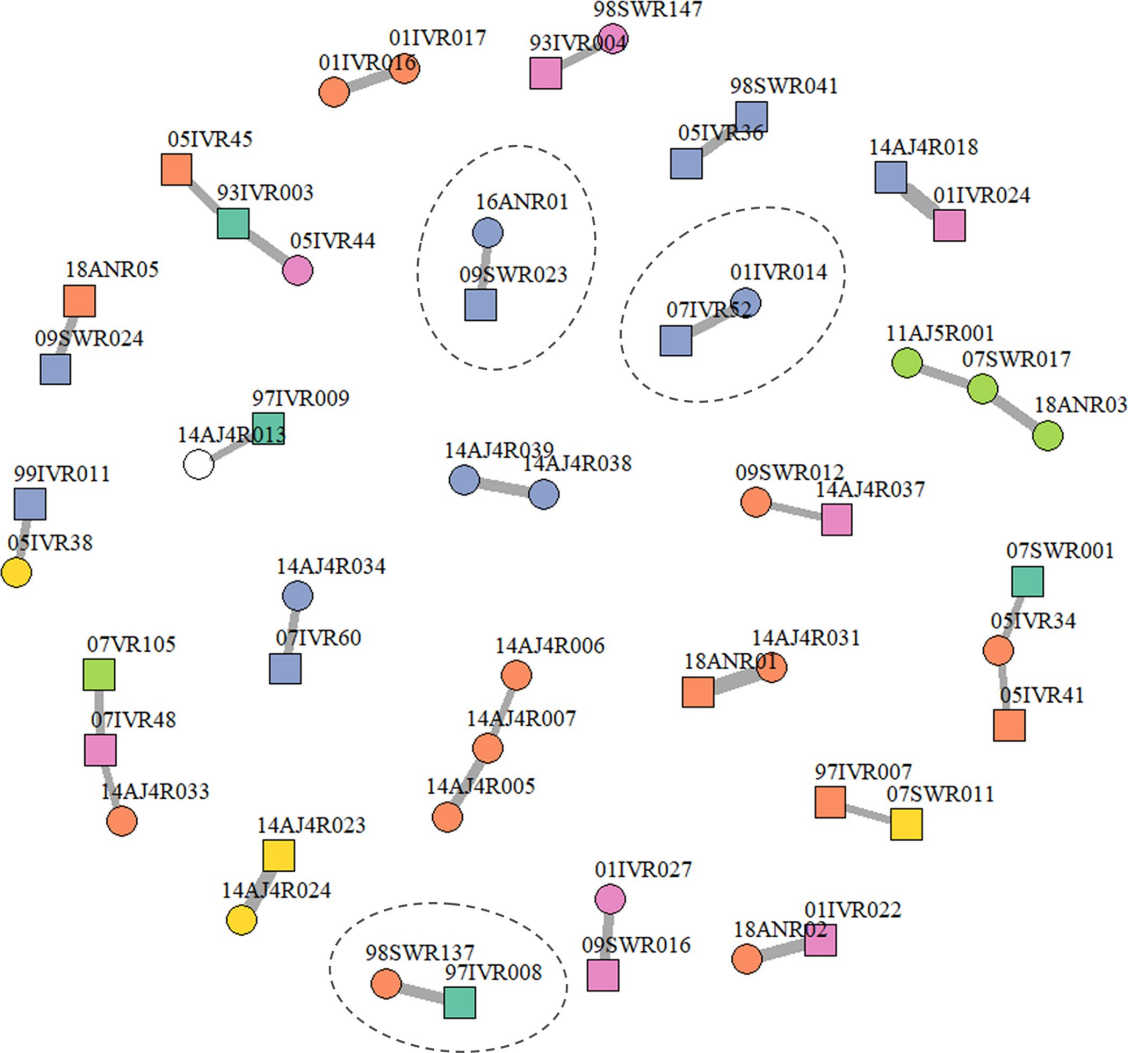
Schematic representation of the dyads and triads in Table 2 (LOD_PO_>6). The strength of evidence (LOD_PO_) is indicated by the width of the line connecting related individuals. Mitochondrial DNA haplotypes are shown by color; circles denote females and squares denote males. The dashed circles show dyads inferred as full-sibling pairs by maximum likelihood.

Fig 3 visualizes the information in Table 2. Triad relationships in this figure result from one individual occurring in more than one dyad. Dyads encircled by a dashed line show possible full-sibling pairs. There were two triads comprising only female animals (Table 2, Fig 3), the first involving 14AJAR006, 14AJAR007 and 14AJAR005 and the second involving 07SWR017, 11AJ5R001 and 18ANR03. The strongest support was for the latter triad. The two pairwise LOD scores were LOD_PO_ = 8.04 and LOD_PO_ = 9.31, also both dyads were related through the same female (07SWR017). The mtDNA haplotype shared by this whale and two females (11AJ5R001 and 18ANR03) had a low population frequency (0.056) which supported the conclusion that the three samples were comprised of either: (1) mother (07SWR017) and her daughters; or (2) a grandmother, her daughter (07SWR017), and her grandchild. It should be noted that deciding which case is appropriate requires age data, which was not available for this study. Also, body length information, which can be used as alternative to age, was not available for this case.

There were three triads comprising two males and one female (Fig 3). Among them, the strongest support was for the triad 93IVR003 (male), 05IVR45 (male), and 05IVR44 (female). Male 93IVR003 had a dyad with a male 05IVR45 and another dyad with female 05IVR44. The two pairwise LOD scores were LOD_PO_ = 7.73 and LOD_PO_ = 8.69, respectively (Table 2). The fact that 93IVR003 had already reached 13.7 m in body length in 1993 indicates sexual maturity. This supports the possibility that the other two animals in the triad (05IVR45 and 05IVR44) are the son and daughter of male 93IVR003, respectively.

### Elapsed time

**Fig 4:**
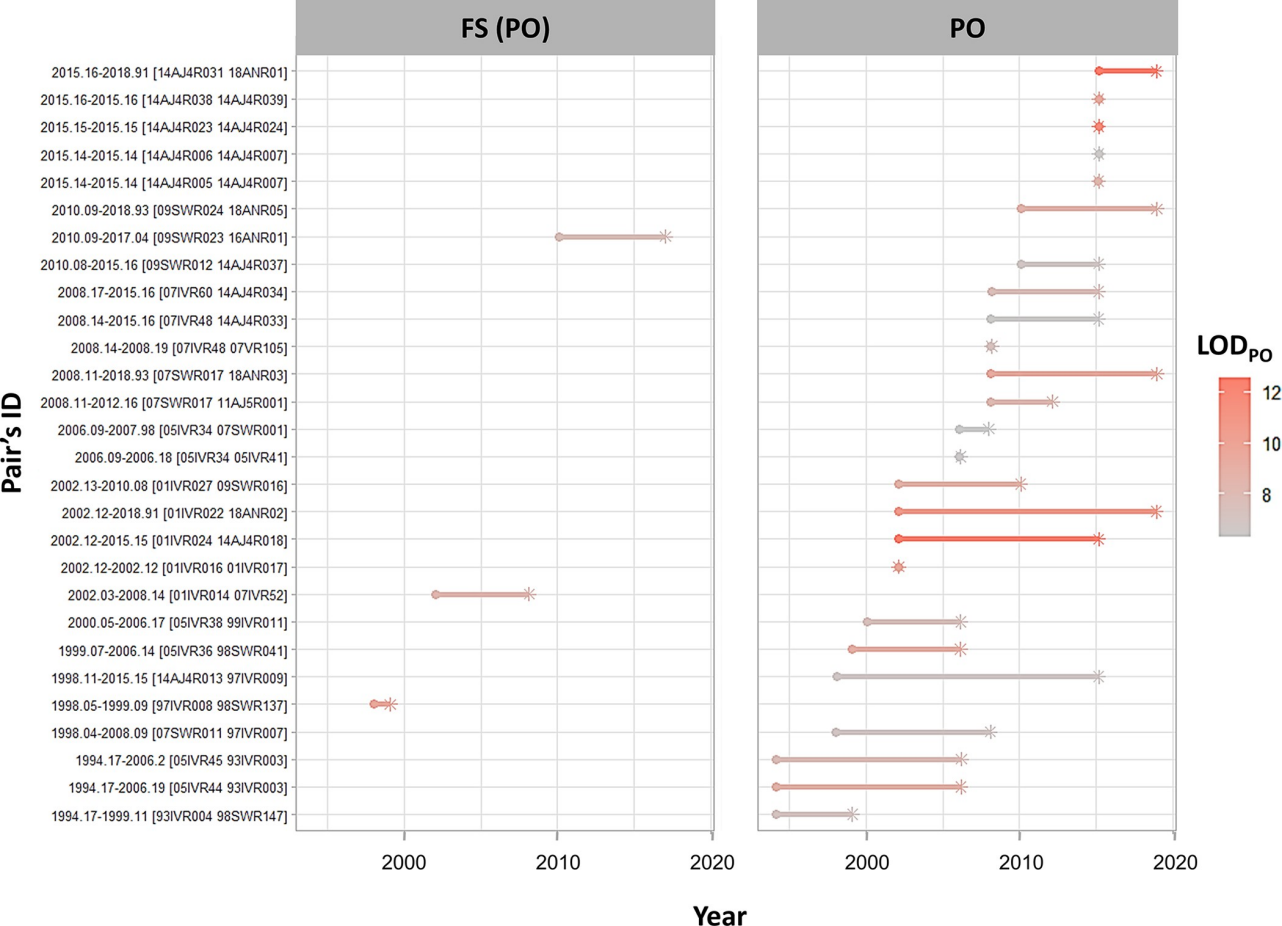
Time intervals between detection of the first and second individuals of dyads with LOD_PO_ > 6 in each assignment category. The square brackets in the y-axis show individual IDs for each pair. Dots and asterisks indicate the first and second encounters, respectively. Color represents the probability of close-related relationship inference for each dyad LOD score (the brighter the color, the higher the score).

Fig 4 shows the elapsed time for dyads with LOD_PO_>6. The maximum time was 17 years (pair 14AJ4R013-97IVR009). There were seven pairs composed of two individuals sampled in the same year, and four of these were recorded as mother-calf pairs by visual observations during the field work (Table 2). The elapsed time for dyads with LOD_PO_<6 is shown in S1 Fig.

### Geographical location of close kin dyads

**Fig 5:**
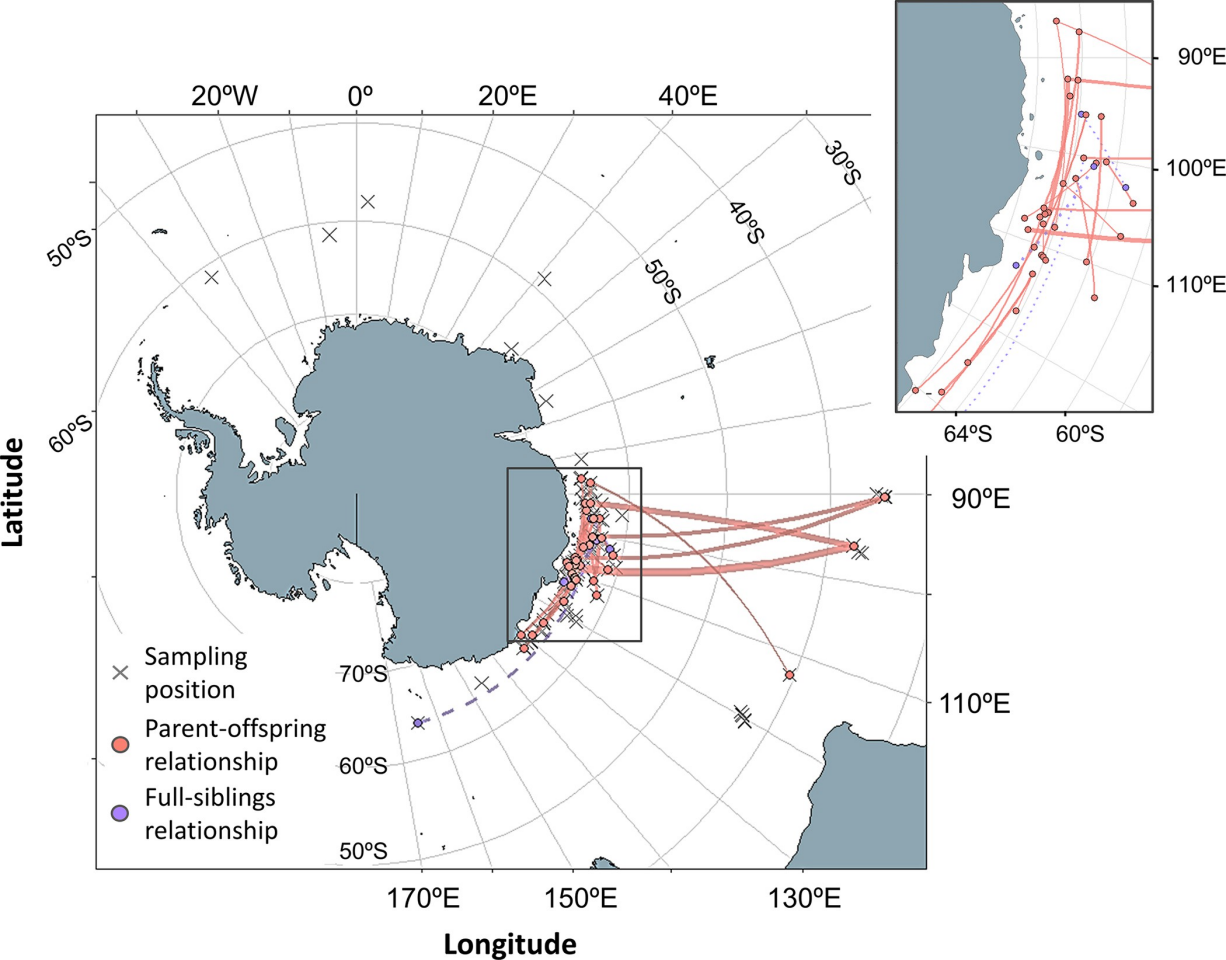
Geographical connections for dyads with LOD_PO_>6 indicating the degree of strength of the connection. The inset shows a map focusing on the Indo sector of the Antarctic where most observations are concentrated. Maps were obtained by R [6] using *rnaturalearth* package [7].

Fig 5 shows the geographical locations and connections for the 28 dyads with LOD_PO_ > 6. Six of these dyads were individuals sampled at low and high latitudes; the high-latitude individuals of these dyads were mainly concentrated between 85°E and 110°E. Within the Antarctic, parent-offspring relationships were concentrated within the Indo sector. There was a single connection between the inner and outer Indo-Pacific sectors through one possible full-sibling relationship. In addition, there were no close kin relationships identified between individuals distributed west and east of 85°E.

### Relationship between LOD score and geographical distance

For these analyses the seven cases of mother-calf pairs observed in field were excluded (Tables 2 and S2). The Wilcoxon test did not find a significant difference in Δ longitude between the PO and unrelated groups, neither for the full data set (*p* = 0.90) nor for the Indo-Pacific sector (*p* = 0.20). Hence, such a comparison did not provide any evidence that close kin aggregate spatially.

**Fig 6:**
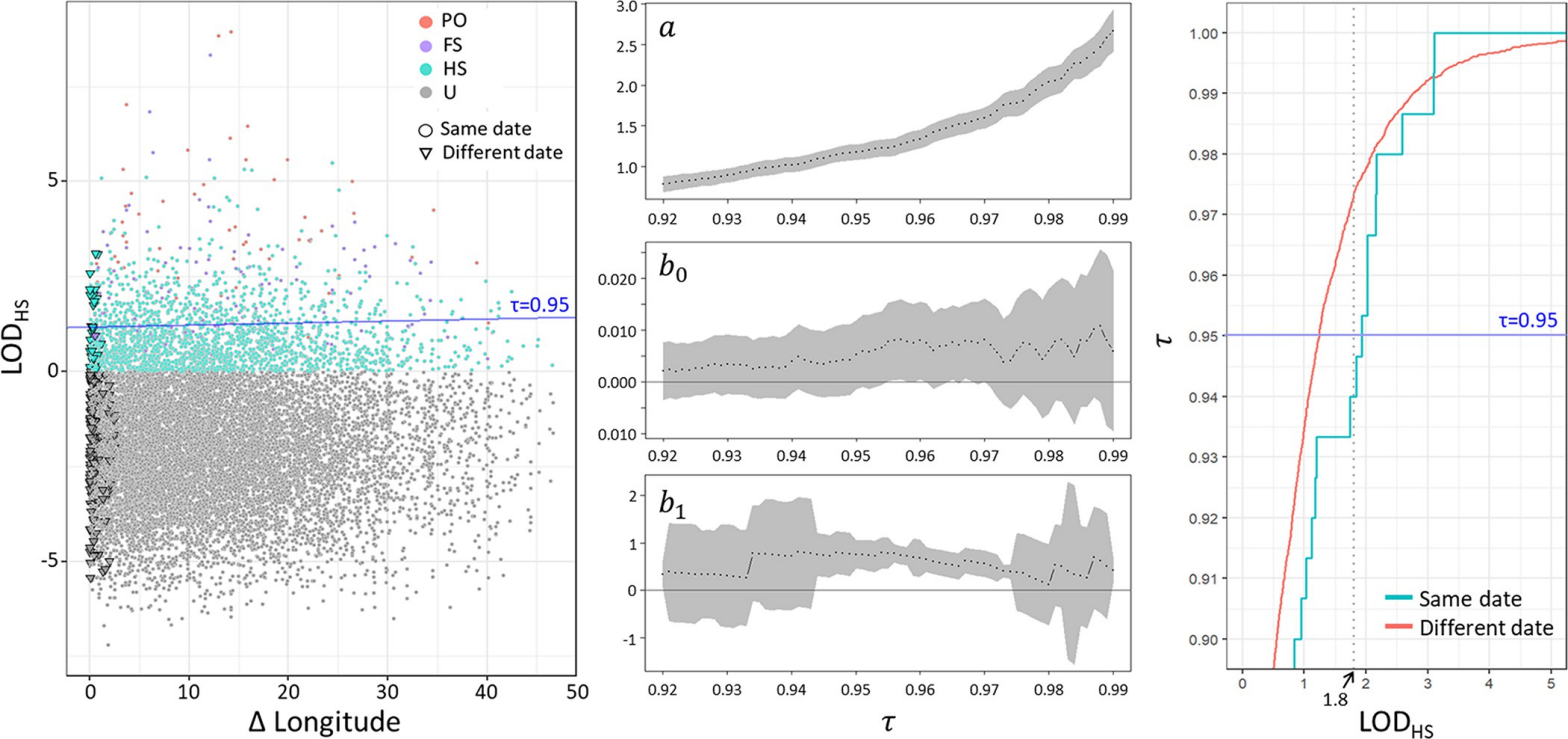
Quantile regression of LOD_HS_ for the Indo-Pacific sector (80°E to 135°E). Left: Data to which the quantile regression is fitted. Each point represents a pairwise comparison, and the symbol ∇ represents pairs that satisfy the criterion for SameDayPos. Relationship type is indicated by color, but is not used in the quantile regression. Fitted regression lines at selected quantile levels (τ) are superimposed on the figure. Middle: Estimated regression coefficient with 95% confidence interval (grey) for different values of τ. Right: A comparison of cumulative distribution function LOD_HS_ for the two levels of SameDayPos. The dotted line shows the position of the median for the simulated LOD_HS_ distribution for the HS-type relationship.

As a second step, we performed linear quantile regression with LOD_HS_ as the response and Δ longitude and SameDayPos as explanatory variables for the Indo-Pacific sector (80°E to 135°E). Fig 6 (left) shows a plot of LOD_HS_ versus Δ longitude, which are the data to which the quantile regression is fit. Regression lines at selected quantile levels are also plotted, but the estimated slope parameter was not significantly different from zero at any quantile level τ (Fig 6, middle column). The estimated effect of SameDayPos was, however, significantly larger zero than in a region around τ = 0.95 (Fig 6, b_1_ in middle column). The same effect can be seen by comparing the cumulative distribution of LOD_HS_ for a range of LOD values that corresponds to a region around τ = 0.95 (Fig 6, b_1_ in right column). The increase in LOD_HS_ seen at this quantile level in the SameDayPos sample coincides with the LOD_HS_ peak expected from half-sibling type relatives in the population. The simulated LOD_HS_ scores (Fig 2, bottom left) for the HS sample has median value at LOD_HS_ = 1.8, which is within the quantile range where we see the increase in LOD_HS_ of the SameDayPos sample compared to that of other pairs. We do not see a similar signature from PO relations in the SameDayPos sample, which would be expected with a center around LOD_HS_ = 5.2, if there were additional PO pairs not detected by direct observation.

The interpretation of this is that pairs of individuals sampled close in time and space on average have a high LOD score, i.e. are more closely related. When this effect is taken into account, there is not additional information in kinship provided by Δ longitude.

## Discussion

To our knowledge, this is the first study using genetics to investigate close kinship in southern right whales in the Antarctic although a paternity assignment study was conducted previously in New Zealand for this species [46]. As mentioned earlier, the identification of close kinship has the potential to contribute to the conservation of this species through the monitoring of important population parameters, which can be estimated from such data. Results of this study are discussed in three sections, the first addressing technical aspects of the analytical approach used to determine close kinship, the second focused on the interpretation of the results in the context of stock structure, movement and site fidelity, and the third focused on the potential use of close kinship data for estimating abundance and some other demographic parameters.

### On the analytical approach used for detecting dyads of close relatives

The inference of close kinship in this study was based on microsatellite DNA genotyping and the index of relatedness (LOD scores) [47]. Under this approach, two individuals may be classified as related if their LOD score exceeds a predefined critical value (the cut-off point). Subsequently, the type of relationship category will be determined by maximum likelihood.

There are several issues relevant to the analytical approach used in this study. One of them is the way of setting of a cut-off point that will maximize the number of correctly inferred dyads of close relatives while keeping the number of dyads incorrectly inferred as close relatives to a reasonable minimum. Another issue is the genotyping error inherent to microsatellite DNA genotyping, on which the detection of close kin dyads is sensitive. A single typing error can render two DNA profiles incompatible with a close kin relationship. Also, the power of the analysis in the context of the number of loci used is relevant for detecting close kin dyads. The more distantly related individuals are, the harder to detect them from among unrelated relationships [28]. Therefore, a large number of loci will provide additional power to the analyses for detecting distantly related individuals.

The choice of the cut-off point on the LOD score should be set depending on the objectives of identifying dyads of close relatives, for example very close or more distant relatives. Finding a LOD cut-off point high enough that false positives can be ignored is a simple and highly conservative step. The 25 PO pairs, including the triad, were finally determined in the present study considering a LOD_PO_ >6. This cut-off point was determined in consideration of the estimation of the false positive rate derived from genotyping error, and on the statistical power for detecting close related individuals. As expected, the dyads determined by using the cut-off of LOD_PO_ >6 included mother-calf pairs observed at the field, supporting the appropriateness of this cut-off. On the other hand, some dyads with LOD_PO_ <6 showed inconsistency with other kind of data. Among these cases, there were three mother-calf pairs identified at the field and by the genetic data (mtDNA) that were listed with LOD_PO_ <6 (see S2 Table). These cases suggest that the determination of the cut-off of LOD_PO_ >6 was somewhat conservative.

The markers are not informative enough to identify individual half sibling pairs with any certainty, and it is therefore not possible to find a meaningful cut-off value the LOD_HS_ to identify individual HS relationships. For a cutoff value LOD_HS_ >3, which is above the median of the LOD_HS_ distribution at 1.8, the expected number of false positive true positives is higher than the observed number classified as HS (Table 1). This shows that our genetic markers are not sufficiently informative to identify individual HS pairs. Nevertheless, LOD_HS_ is useful as an index of relatedness; to infer directly about which pairs are half siblings, more markers are needed. Although the individual HS pairs are not directly identifiable, the quantile analysis reveals a higher number of close kin among the individuals sampled close in time and space.

The uncertainty for the inference of close kin dyads is related mainly to the amount of information in the DNA profiles, but also to genotyping error. In the present study, a simple error model with a constant and independent per-allele error rate was used based on genotyping error information from the North Pacific right whale [31]. Such information is considered appropriate because the North Pacific right whale is a sister species, and because the laboratory work for both species was carried out at the same laboratory. In any case, in the present study a sensitivity test in which the genotyping error rate was doubled was performed and confirmed the robustness of the inference. On the other hand, the present study relied on microsatellite DNA markers. The number of loci and their diversity are important for detecting close related relationships, and as the number of loci and diversity are getting higher, the inference feasibility and accuracy are higher. For increasing power and diminishing uncertainty there are roughly two candidate strategies in the future, one is increasing the number of microsatellite markers for the available (and new) DNA samples, and the other is the use of different nuclear markers, e.g., single nucleotide polymorphism (SNPs). Unfortunately, some of the samples of southern right whales are no longer available.

Uncertainty associated with the analytical approach used in this study (outlined above) could be diminished by the use of other genetic markers as well as biological information such as sex, age and body length of the individuals. In the present study, all this information, apart from age data, was used and proved to be very useful for assisting in the interpretation of the close kin dyads determined by microsatellite DNA and the LOD approach. It is strongly recommended that such information be obtained and included in future studies on inference of close kinship. Photogrammetry studies based on drone surveys could provide more precise information on the body length of individuals.

The approach used in this study to determine close kinship has advantages regarding other methods such as records of natural markings. The latter method is not sufficient to identify young individuals and natural markings are not reliable in some species.

Inference of close kinship relationships based on the LOD score revealed that such relationship occurred in a particular area, which is associated with a particular population of this species in the Southern Hemisphere. Also, results were clear to indicate a relationship between individuals sampled at the same day and by the same vessel, while separating distance (delta longitude) is not informative.

### Interpretation of results in the context of stock structure, movement and site fidelity

Previous studies on distribution and movement based on photo-id matches [24], historical whaling data and recent sighting surveys [22] showed that whales in the Indo sector of the Antarctic are associated with breeding grounds in the Australasian regions. Furthermore, a mtDNA analysis showed significant genetic differences between whales in the Indo sector of the Antarctic (85°-135°E) and whales from breeding grounds in Argentina, South Africa and New Zealand but not with whales from Southwest Australia (Pastene *et al*., 2018 [Unpublished]). This result suggested a close relationship between whales in the Antarctic Indo sector and whales in the Southwestern Australia breeding ground. Close kinship information strongly supported the view that whales in the Indo sector of the Antarctic belong to a stock related to Southwestern Australia as most of the dyads were found within the sector 85°E-135°E (within and between austral summer seasons), and between this Antarctic sector and whales in lower latitudes off Southwestern Australia (between different austral summer seasons). In contrast, no close kinship relationships were found between whales within and outside of this longitudinal sector, with the exception of a close kin pair where one of the pair was located between 160° and 170°E. It is interesting to note that sighting surveys have been conducted systematically in the sector between 35°E and 145°W in austral summer and that concentration of southern right whales were nevertheless observed only in the sector 85°-135°E [48].

Results of this study support the hypothesis that fidelity to feeding areas is inherited from mother to offspring (see [49,50]), given that most of the maternally-related parent offspring pairs occurred in a similar sector in the Antarctic (85°-135°E) or represented connections between low and high latitude waters in this longitudinal sector.

As mentioned above, a possible full-sibling pair was found between the Indo sector and the sector between 160° and 170°E. This can be explained by the sporadic dispersion of close kin whales of the Southwest Australia stock in the core longitudinal sector (85°-135°E) into a more eastern longitude. Southern right whale sightings in the sector 135°E and 145°W are extremely rare despite considerable sighting effort applied as mentioned above. It seems that whales from the genetically-differentiated New Zealand breeding ground do not migrate south as in the case of the whales from the Southwestern Australia breeding ground. An alternative explanation is that whales from different breeding grounds, e.g., Southwest Australia and New Zealand breed (e.g., [51]) and that their ‘hybrid’ offspring migrate to different sectors of the Antarctic. However, we do not have any evidence of monogamy, so full-siblings can be expected to be rare. A third interpretation is the possibility of a false-positive dyad for this particular case.

Four of the five close kin dyads were found between the Antarctic and offshore areas off Southwestern Australia (Fig 5). The four whales in lower latitude waters were sampled in December 2018, and the other members of these close kin dyads were sampled in previous years (2002, 2008, 2010 and 2015) in Antarctic waters. All whales were sampled during the austral summer. It is unknown whether the whales feeding in offshore waters in December 2018 fed at a different feeding area in a later period of that season. Also, it is unknown whether the whales feeding in the Antarctic in February of the four different years above had fed at a different feeding area earlier in those seasons. What is clear is that closely related whales (PO pairs) could feed in different areas, and this finding is consistent with the conclusion of Mackay et al. [25] of three probable foraging grounds for the animals tagged in the Australasian wintering grounds. Site fidelity of southern right whales to the feeding grounds in the Antarctic was studied based on re-sampling of genetically-identified individuals [26]. These authors examined a total of 153 genetic samples of southern right whales collected in the Indo sector of the Antarctic, south of 60°S in the period 1993/94-2015/16. They found that at least some females (n = 4) and males (n = 4) returned to the same feeding ground south of 60°S in multiple seasons. They concluded that more definitive inferences on site fidelity and movement ranges will require a larger number of biopsy samples, from both south and north of 60°S. The same recommendation applies to future studies on close kinship inference.

### Potential use of close kinship for estimating abundance and demographic parameters

Close kinship data from this study can be used for estimating the abundance of the stock using close-kin mark-recapture (CKMR) methods [27]. The CKMR method is an extension of classical mark-recapture methods. In previous applications of this method to fish species, age data have been available, which simplified the calculation of kinship recapture probabilities. No age data is available for southern right whales so if the CKMR method is applied to this species in the future, some modification of the method that covers for the lack of age data, will be required (e.g., [47]).

Most of the studies on the biology and abundance estimates of southern right whales have been focused so far on calving grounds in low latitude waters, and limited studies of such kind are available for feeding grounds in the Antarctic. Comprehensive studies involving both low latitude breeding areas and high latitude feeding areas are required for developing sound policies on conservation of southern right whales in the Southern Hemisphere. The present novel study on close kinship in this species was conducted in the Indo-Pacific sector of the Antarctic, which has been suggested as one of the feeding grounds of the Southwest Australian population. New information on links between low and high latitudes areas of this population was provided in this study as well on the longitudinal range of distribution of this population in the Antarctic feeding ground in summer. As mentioned earlier close kinship data are potentially useful for estimating some demographic parameters, which should be monitored for conservation purposes. Furthermore, the information on close kinship in this study could also be informative about inbreeding in this species. However, the question about which population one is estimating the size of, when using feeding ground data, requires careful consideration, and methodological developments if the aim is to estimate the size of the breeding population.

## Supporting information

S1 Fig Time intervals between detection of the first and second individuals of dyads with LOD_PO_ < 6 in each assignment category. (TIF)

S1 Table Overview of the DNA samples for southern right whales collected in the Antarctic Ocean during dedicated sighting surveys in 1993–2019. (XLSX)

S2 Table Summary of information for the dyads (D1, D2) identified with LOD_PO_ < 6. (XLSX)
