# Supplementary figures and images for: Geographical distribution of close kin in southern right whales on feeding grounds
Source: PLoS One. 2024 Apr 25;19(4):e0301588. doi: 10.1371/journal.pone.0301588 (PMC11045074; doi:10.1371/journal.pone.0301588)

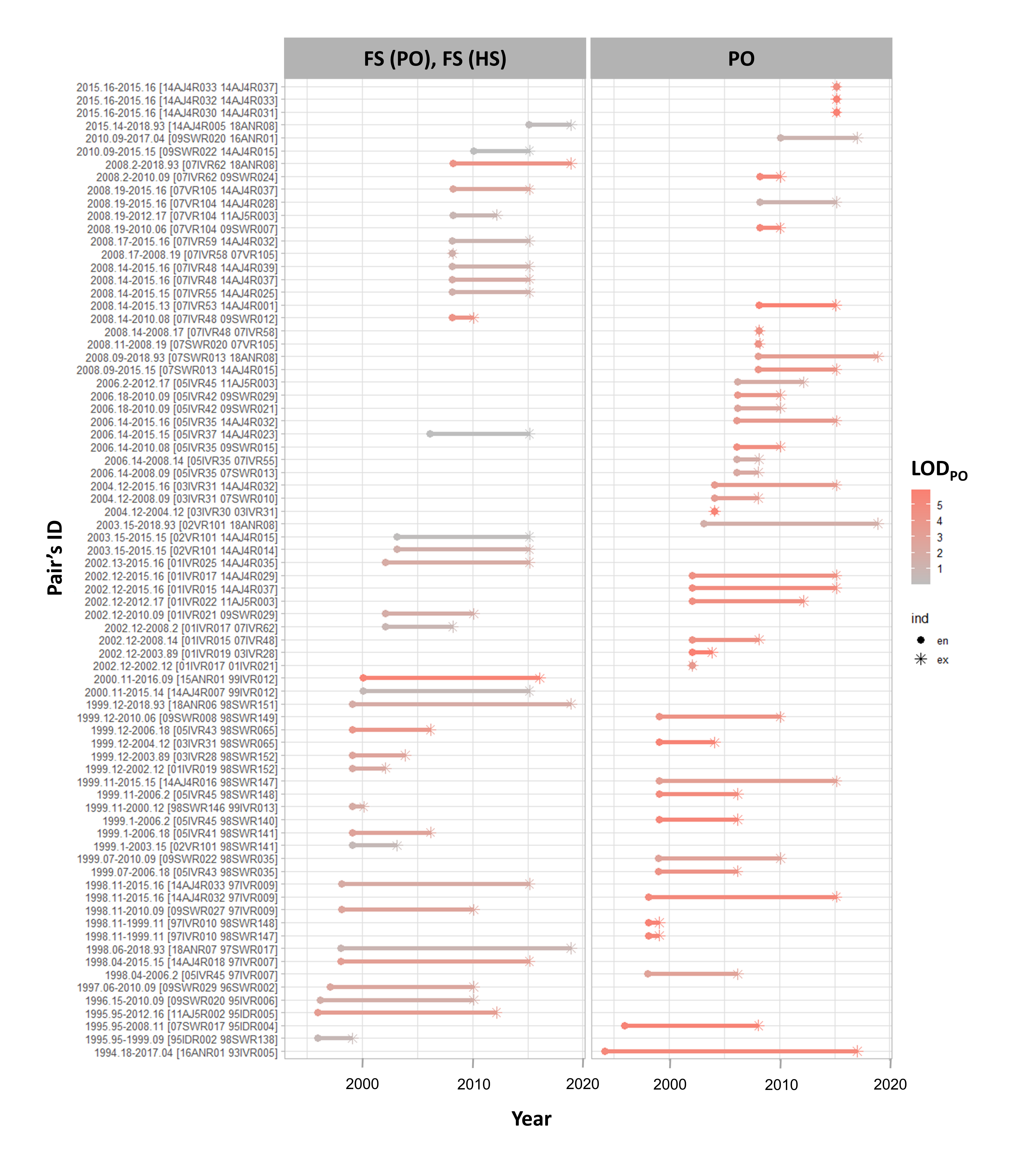

Supplement: S1 Fig — (TIF) [file pone.0301588.s001.tif]
